# PersonMe: A clinical trial for Personalised Psychiatry from your Sofa

**DOI:** 10.1192/j.eurpsy.2026.11858

**Published:** 2026-07-31

**Authors:** E. Van Assche, B. T. Baune

**Affiliations:** 1 Department of Psychiatry, University of Münster, Münster, Germany; 2 Melbourne Medical School, Department of Psychiatry; 3 The Florey Institute of Neuroscience and Mental Health, University of Melbourne, Melbourne, Australia

## Abstract

**Introduction:**

Personalized psychiatry often entails a more intensive treatment paradigm and follow-up, typically requiring more personal resources. However, due to a growing shortage of psychiatrists, nurses, and others, many regions and countries will need to adopt a resource-optimized care model that incorporates personalization within an ambulatory care framework.

**Objectives:**

We present an innovative, ongoing clinical trial that tests the treatment concept of ‘all-inclusive’ personalized medication dosing at home.

**Methods:**

After discharge from the hospital or during ambulatory care, patients are monitored for two blocks of two weeks over a 12-week period, using movement sensors and experience sampling to detect changes in behavior and movement that indicate side effects or an increased relapse risk with the current medication. If any indicators of suboptimal dosing are detected by the secured platform via the tracking devices (research-optimized and regular consumer devices e.g., apple watches), patients are prompted by the platform to send a dried blood spot to measure medication plasma levels, taking into account the time since the last dose. The same platform is used to inform the patient within 24 hours if any action is necessary regarding a dose adjustment, for which the patient is provided with an appointment suggestion. Patients can choose to extend the tracking beyond 12 weeks. If no issues arise as indicated by the platform, the patient will have contact with a doctor only every three months. This model highlights patients at risk for relapse or discontinuation due to side effects, all from the comfort of the patient’s sofa.

**Results:**

The project implements multiple novel aspects relevant to personalized psychiatry. We test a highly innovative model that relies on experience sampling and digital phenotyping in an ambulatory setting. In addition, we evaluate therapeutic drug monitoring (TDM) in an ambulatory setting. The project also encompasses the development of new algorithms that allow the continuing learning from patient data to be used within the patient to prompt warnings if changes are detected. Both interventions and the integration of all information within the algorithm increase the accessibility of personalized psychiatry for ambulatory care. Successful implementation also opens up opportunities for rural and less care-dense areas.

**Conclusions:**

Overall, this clinical trial tests multiple innovative elements in a unique and patient-oriented combination, preparing ambulatory care for the future. The development of personalized applications and testing their application and implementation in real-live personalized patient care through an innovative clinical trial, allow for the urgently needed first-hand experience with extensive digital phenotyping. The aim of the study is to show that digital phenotyping for psychiatry and ambulatory drug monitoring to make personalized psychiatry more accessible, is in reach.

**Disclosure of Interest:**

None Declared

